# The rationale for treating uveal melanoma with adjuvant melatonin: a review of the literature

**DOI:** 10.1186/s12885-022-09464-w

**Published:** 2022-04-13

**Authors:** Anna Hagström, Ruba Kal Omar, Pete A. Williams, Gustav Stålhammar

**Affiliations:** 1 Department of Medicine, Karolinska Institutet, D1:04, 171 76 Stockholm, Sweden; 2 Department of Clinical Neuroscience, Division of Eye and Vision, St. Erik Eye Hospital, Karolinska Institutet, 171 64 Stockholm, Sweden; 3 St. Erik Eye Hospital, Box 4078, 171 04 Stockholm, Sweden

**Keywords:** Uveal melanoma, Choroidal melanoma, Melatonin, Adjuvant treatment, Survival, review, Metastasis, Micrometastasis, Dormancy, Rationale

## Abstract

**Background:**

Uveal melanoma is a rare form of cancer with high mortality. The incidence of metastases is attributed to early seeding of micrometastases from the eye to distant organs, primarily the liver. Once these seeded clusters of dormant tumor cells grow into larger radiologically detectable macrometastases, median patient survival is about 1 year. Melatonin is an important hormone for synchronizing circadian rhythms. It is also involved in other aspects of human physiology and may offer therapeutic benefits for a variety of diseases including cancer.

**Methods:**

Articles involving the physiological effects of melatonin, pharmacokinetics, and previous use in cancer studies were acquired using a comprehensive literature search in the Medline (PubMed) and Web of Science databases. In total, 147 publications were selected and included in the review.

**Results:**

Melatonin has been observed to suppress the growth of cancer cells, inhibit metastatic spread, enhance immune system functions, and act as an anti-inflammatory in both in vitro and in vivo models. Melatonin may also enhance the efficacy of cancer treatments such as immuno- and chemotherapy. Numerous studies have shown promising results for oral melatonin supplementation in patients with other forms of cancer including cutaneous malignant melanoma. Cell line and animal studies support a hypothesis in which similar benefits may exist for uveal melanoma.

**Conclusions:**

Given its low cost, good safety profile, and limited side effects, there may be potential for the use of melatonin as an adjuvant oncostatic treatment. Future avenues of research could include clinical trials to evaluate the effect of melatonin in prevention of macrometastases of uveal melanoma.

## Introduction

Uveal melanoma is the most common primary intraocular malignancy in adults and is associated with a high incidence of metastasis, a high mortality rate, and poor response to current treatments [1–3]. The cancer originates from uveal melanocytes and possesses several features seen in other melanocytic tumors of the central nervous system (CNS) [4]. Uveal melanoma is rarely present in children and the incidence increases with advancing age. Some studies suggest that uveal melanoma has a higher incidence rate in males over the age of 65, while other studies showed no sex predilection [5–7]. Strong evidence for incidence variation between different ethnic groups exists, where non-Hispanic white individuals have the highest incidence, with an overall non-Hispanic white:black incidence ratio of 18:1 [8]. Incidence rates for cases per million per year are 5.1 in the USA (1973–2008) and 1.3–8.6 in Europe (1983–1994) with generally increasing incidence from southern to northern regions [9, 10]. No major differences in survival have been observed when stratifying cumulative uveal melanoma-related mortality rates by year of diagnosis [11]. The estimated five, ten, 15, 20, 25 and 30-year relative survival rates are 79, 66, 60, 60, 62 and 67%, respectively [12].

Click or tap here to enter text.

There are two predominant types of risk factors for uveal melanoma: genetic and environmental. There are several low-risk genetic loci which include several single nucleotide polymorphisms (SNPs) in genes encoding pigment proteins (*HERC2*, *OCA2*, *IRF4*) as well as other genes including *TERT* which encodes for telomerase reverse transcriptase, and *CLPTM1L*, which encodes cleft lip and palate transmembrane protein 1-like protein. High-risk loci have been identified in the genes *BAP1*, *MLH1*, *PALB2*, and *SMARCE1* [13]. Other risk factors include atypical or common cutaneous nevi, welding, occupational cooking, fair skin color, light eye color, sunburn, iris nevi, and cutaneous freckles [14]. The quantity and quality of melanin appears to play an important role in uveal melanocytes, where there are two types of melanin; black to brown eumelanin, and yellow to red pheomelanin. Eumelanin is associated with dark eyes while pheomelanin is associated with light eyes and it has been suggested that eumelanin acts as an antioxidant while pheomelanin acts as a pro-oxidant which may help explain its correlation to a higher incidence of uveal melanoma [15].

Inflammation has been suggested to play a key role in the development of uveal melanoma with high numbers of CD68+ macrophages being associated with heavy pigmentation, microvascular density, epithelioid cells, and increased 10-year uveal melanoma-related mortality rate [16].

The most common cytogenetic aberrations associated with uveal melanoma include monosomy of chromosome 3 and an isochromosome of 8q [17]. In turn, monosomy 3 is associated with mutations in the tumor suppressor gene *BAP1*, located on the short arm of chromosome 3 and is associated with uveal melanoma [18]. In the absence of *BAP1* mutations, other gene mutations are usually present, such as *SF3B1*, a gene associated with late-onset metastasis and *EIF1AX*, associated with low-risk profile of uveal melanoma and a low risk for metastasis [19, 20]. Somatic mutations in *GNAQ* and *GNA11* subunits of G_q_-protein have been correlated to uveal melanoma oncogenesis, but do not correlate with metastatic risk [21]..

Primary uveal melanoma is typically treated by enucleation or eye-preserving plaque brachytherapy. Despite treatment, metastatic rates reach 30 to 50% even with removal of the eye due to the early seeding of micrometastases [22, 23]. These treatments result in similar survival rates for medium-sized tumors, which are defined as tumors between 2.5 mm and 10.0 mm in height, though no more than 8.0 mm in height whenever the tumor is near the disc, and no more than 16.0 mm in diameter [24].

Tumor cells migrate from the intraocular tumor via a strictly hematogenous route to the systemic circulation from which they can extravasate and establish micrometastases (colonies of tumor cells that are too small to cause symptoms or to be detectable by radiological examinations), primarily in the spleen and liver [25]. The latter is the most common site for metastasis, which may be related to the affinity of cMET-ligands by uveal melanoma-cells to HGF-receptors of hepatic stellate cells [26]. Estimations based on tumor doubling times indicate that the seeding of metastases occurs well before detection of the average primary tumor [27, 28]. The micrometastases can remain dormant in their metastatic niche for years or decades before switching to proliferation and macrometastasis [29]. At that point, mean patient survival is ~ 1 year, with low response rates to immune- and chemo- therapy [13]. As such, additional adjuvant treatments for uveal melanoma and their metastases are of great therapeutic need.

## Aims

Melatonin has been studied as an oncostatic agent and has shown promising results in breast, prostate, colon, and liver cancers [30–33]. However, research regarding melatonin’s potential therapeutic role in uveal melanoma is limited. This review aims to explore current data surrounding melatonin as an oncostatic agent and whether future clinical studies should be performed to further investigate the effects of melatonin as an adjuvant treatment in uveal melanoma.

## Literature search

Data were acquired with a comprehensive literature search in the Medline (PubMed) and Web of Science databases for peer reviewed published articles that described relevant results. The following search terms were used and matched to appropriate medical subject headings: (“Melatonin” AND (“physiological effects” OR “cancer” OR “animal studies” OR “oncostatic effect” OR “mechanism of action” OR “pharmacokinetics” OR “clinical trial”)). The search strategy was restricted to titles and/or abstracts of human clinical studies published in English. No restrictions based on year of publication were made. The latest search was performed on August 1st, 2021. All available studies were included and could be accessed in full via the University Library, Karolinska Institutet. Trial registries, unpublished studies, grey literature, animal studies, laboratory studies, letters to the editor, correspondence, notes, editorials, and conference abstracts were not considered. Reference lists of included articles were searched for additional studies.

**Fig. 1:**
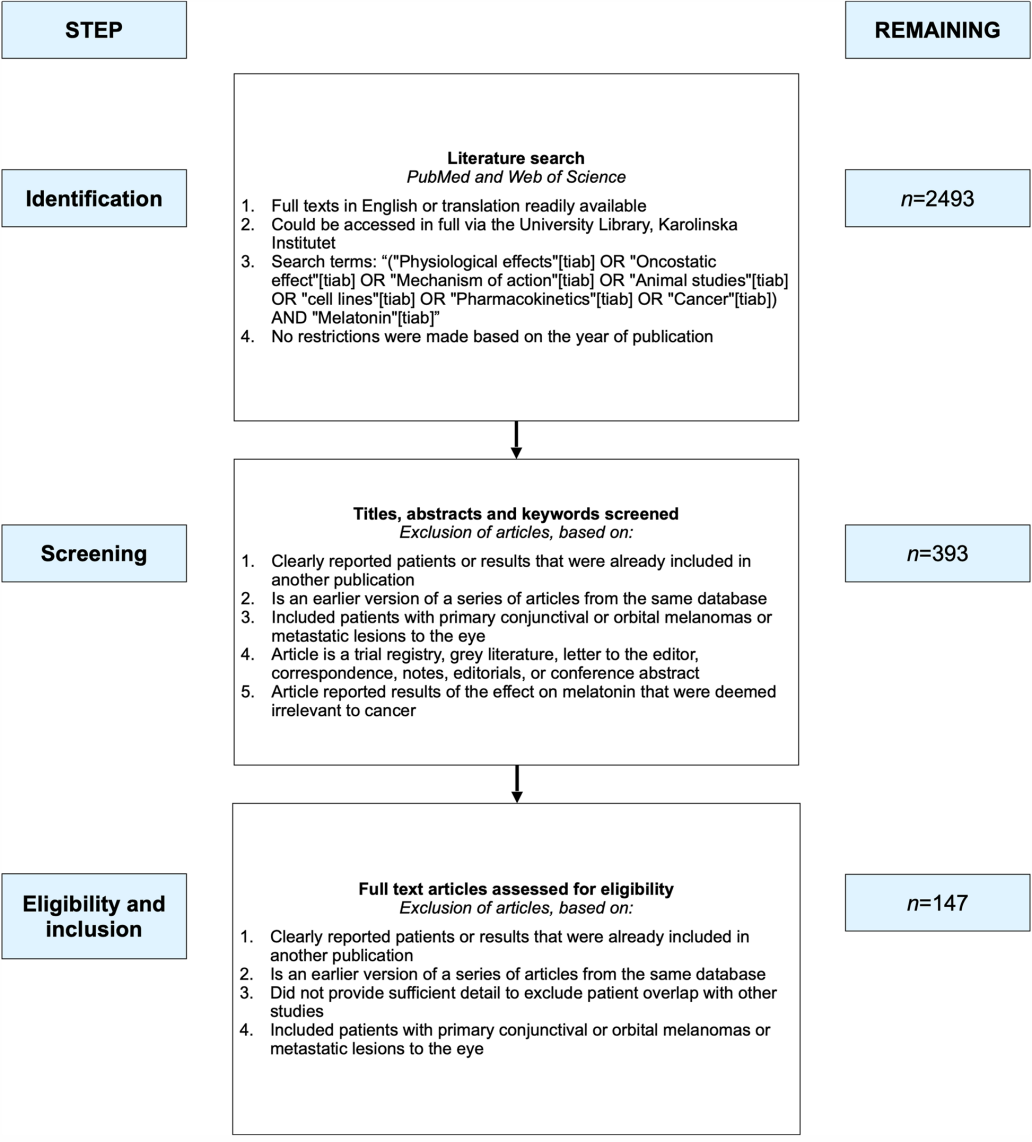
Literature search. The selection of articles for this analysis was performed in three steps. Articles were identified using specific search terms, abstracts were then screened, and lastly, full text articles were assessed for eligibility based on our exclusion criteria. Through this process 147 articles were selected and included in this review

The selection of articles for this analysis was performed in three steps as illustrated in Fig. 1: Identification, title, abstract and full-text screening, and inclusion. Abstract screening of articles identified in the literature search was done independently by the two first authors, with any disagreements resolved by discussion. Publications were included for full-text screening if they reported relevant results. The search string resulted in a total of 1293 publications. After title, abstract and full-text screening, 147 publications remained in this review. Studies were excluded if they 1) were earlier versions of a series of articles from the same database or center or 2) reported patients that were already included in another publication. Studies including patients with primary conjunctival or orbital melanomas or metastatic lesions were not considered. The same inclusion and exclusion criteria applied to full-text screening (if not evident in title or abstract). The preferred reporting items for systematic reviews and meta-analyses (PRISMA) were not followed as this is a narrative review rather than a systematic review or a meta-analysis.

## Review

### Melatonin

Rene Descartes described the pineal gland as the “seat of the soul”. The earliest indications that the pineal gland was biologically active were described ~ 2000 years ago by the Greek physician Galen in his 8th work De Usu Partium — On the Usefulness of the Parts of the Body [34]. Evidence that the pineal gland produced a biologically active substance was published as early as 1917 [35]. Later, this substance was named melatonin, based on the observation that extracts from bovine pineal glands produced a skin lightening response when applied to frog skin. In 1958, Lerner et al. isolated melatonin itself (5 methoxy-N-acetyltryptamine) [36]. Melatonin is involved in numerous biological processes, playing an important role in human physiology, and may offer therapeutic benefits for a variety of diseases. Systemic melatonin is mainly secreted by the pineal gland, however, additional sources include the retina, skin, bone marrow cells, and the gastrointestinal tract [37–41].

**Fig. 2:**
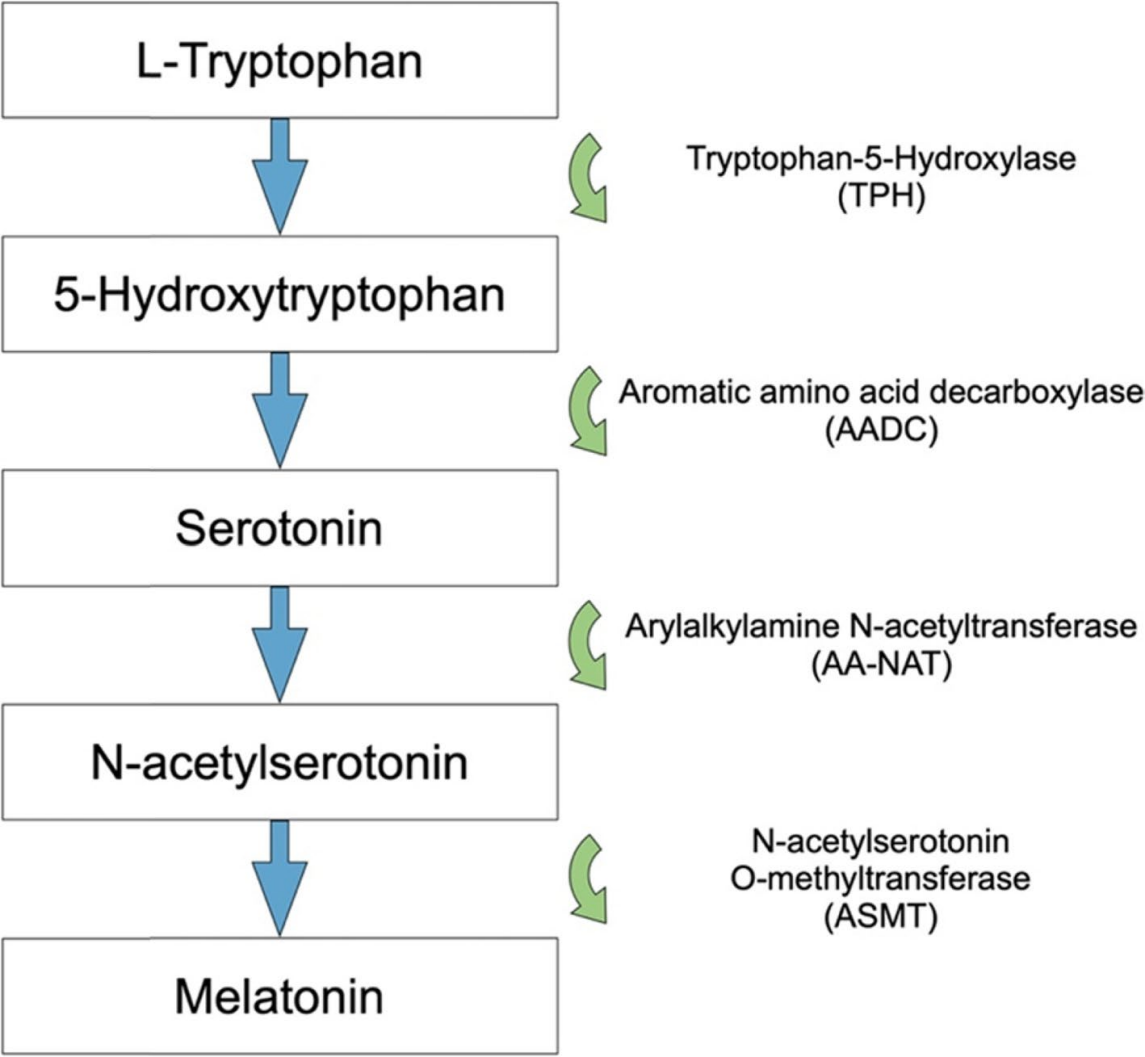
Melatonin synthesis pathway. Tryptophan is hydroxylated by tryptophan hydroxylase (TPH) to 5-hydroxytryptophan which is subsequently decarboxylated by aromatic amino acid decarboxylase (AADC) resulting in serotonin. Serotonin is converted to N-acetylserotonin by Arylalkylamine N-acetyltransferase (AA-NAT) which is then catalyzed by N-acetylserotonin O-methyltransferase (ASMT) to produce melatonin

Melatonin was previously thought to be made in the cytosol, however, more recent research suggests that melatonin is primarily produced in the mitochondria [42]. Melatonin is synthesized from serotonin which in turn is synthesized from the amino acid tryptophan (Fig. 2). In the pineal gland, tryptophan is taken up from the blood and hydroxylated to 5-hydroxytryptophan which is subsequently decarboxylated to serotonin. Serotonin is in turn converted to N-acetylserotonin and then converted to melatonin ready to be released into the blood and cerebrospinal fluid [43–45].

Melatonin is an evolutionarily preserved molecule found in all microorganisms, plants, and animals [46]. Key functions of melatonin include the regulation of circadian patterns, such as fluctuations in core body temperature and sleep-wake cycles, seasonal reproduction, immune system enhancement, and glucose regulation [47, 48]. While melatonin performs several hormonal functions, this unique molecule also exhibits paracrine and autocrine effects and acts as an antioxidant and a free radical scavenger [49–51].

### Endogenous production

**Fig. 3:**
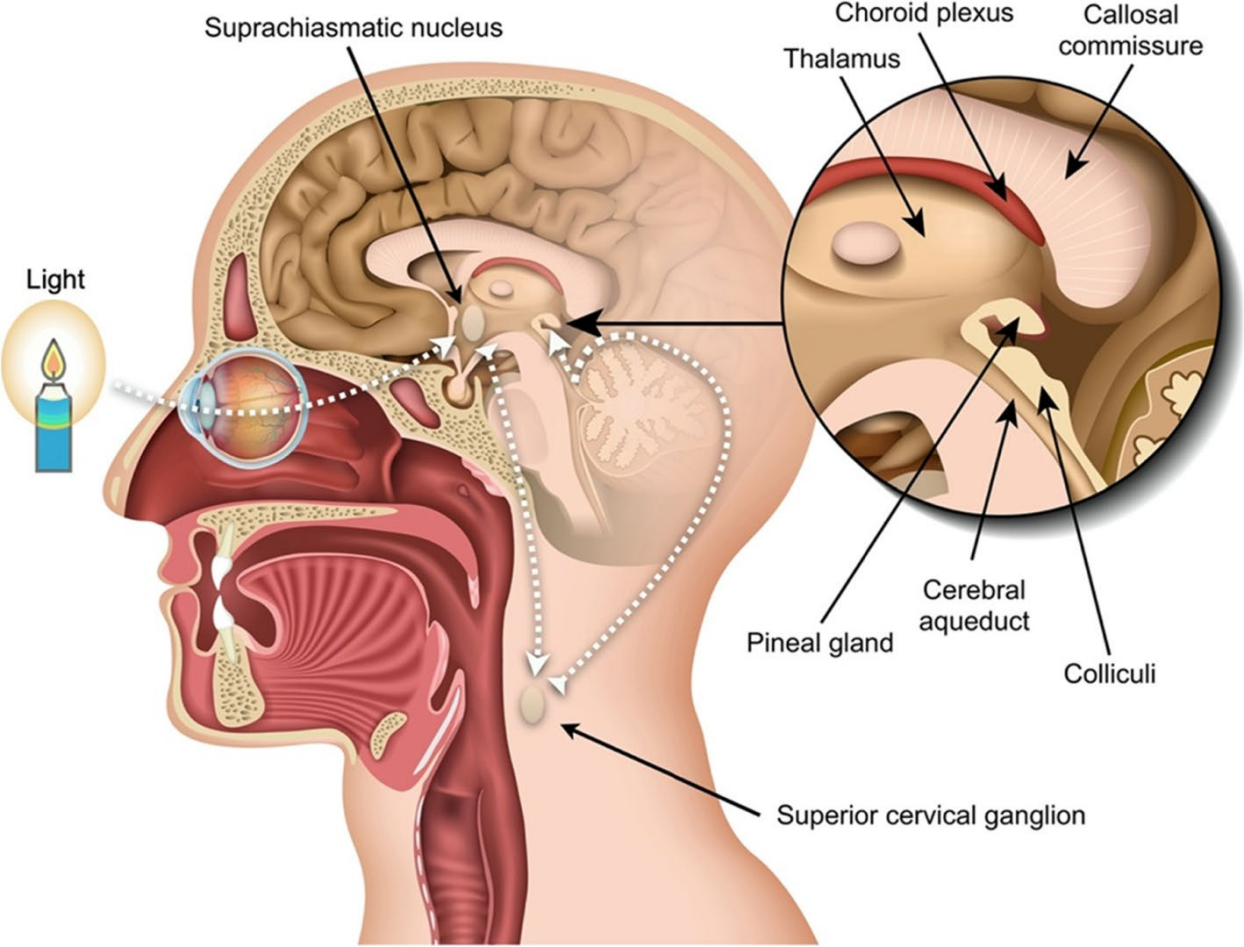
The anatomy of melatonin secretion. The production and release of melatonin are mainly mediated by postganglionic retinal nerve fibers that exits the eye through the optic nerve, pass through the retinohypothalamic tract to the suprachiasmatic nucleus and then to the pineal gland via the superior cervical ganglion. This axis is activated by darkness and suppressed by light. The circadian rhythm of melatonin secretion is to a lesser extent also controlled by the suprachiasmatic nucleus

While melatonin is produced by cells in several types of tissue throughout the body, the primary source of systemic melatonin is the pineal gland. Unlike other endocrine glands, the pineal gland does not store the melatonin it produces but, instead, secretes it directly into the blood and cerebrospinal fluid. From the vasculature, melatonin is distributed across various fluids and tissues including saliva, urine, amniotic fluid, semen, and breast milk [48]. The secretion of melatonin is mediated by light and dark cycles via the suprachiasmatic nucleus (SCN) in the hypothalamus. Light is detected by intrinsically photoreceptive retinal ganglion cells (ipRGC; also named melanopsin sensitive retinal ganglion cells) in the retina which are particularly sensitive to blue light (480 nm), found in natural sunlight as well as in artificial light sources such as LEDs [52, 53]. This information is relayed to the SCN via the retinohypothalamic tract before forming a complex signaling pathway to the paraventricular nuclei (PVN), the intermediolateral nucleus of the spinal cord (IML), the superior cervical ganglion (SCG), and finally, the pineal gland [54]. Production of melatonin takes place at night, in the absence of light (Fig. 3) [41].

During the day, serum concentrations of melatonin remain low (10 pg/ml), however, levels begin to increase after sunset, reaching a peak concentration of ~ 60 pg/ml between 2:00 and 4:00 a.m. [55]. Peak concentrations vary among individuals and have been found to be the highest in children between the ages of one and five, thereafter decreasing with age [56]. Meanwhile, melatonin concentrations have been found to be lower in individuals with certain conditions including depression, Alzheimer’s disease, age-related macular degeneration, idiopathic scoliosis, and autism spectrum disorders [57–61].

When taken orally, a 0.5 mg tablet of exogenous melatonin is equivalent to physiological levels of endogenous melatonin [62]. Exogenous melatonin is commonly taken as a supplement to combat insomnia as well as to ameliorate the effects of jetlag, however, there is increasing evidence suggesting that taking exogenous melatonin may have benefits in a variety of diseases including cancer [63].

### Pharmacokinetics

Melatonin, whether administered orally or intravenously, is absorbed via first-order kinetics. Melatonin administered orally has a half-life of absorption of 6 min. T_max_ of 10 mg orally administrated melatonin was 41 min in a study, which is in agreement with early studies where T_max_ varied between 46 to 65 min when doses ranged between 0.5 to 6 mg. When administered intravenously, the elimination of 10 mg of melatonin was 39 min, which corresponded to early studies where the elimination varied between 28 and 60 min for doses ranging between 0.005 to 2 mg. Melatonin has low bioavailability due to hepatic first-passage metabolism, at about 3% with some inter-individual variability. Other studies have suggested higher bioavailability between 9 and 33% with similar findings in regards to inter-individual variability [64]. The metabolism of melatonin occurs primarily in the liver through hydroxylation by the enzyme CYP1A2 and conjugation with sulfuric or glucuronic acid. Melatonin undergoes secondary metabolism in the kidney and is excreted in the urine as an inactive metabolite; 6-sulfatoxymelatonin (6-SM), which is used as a reliable marker of melatonin production [62, 65].

### Mechanism of action

Melatonin is lipophilic and interacts with a broad variety of receptors including specific membrane receptors, nuclear receptors, intracellular proteins such as calmodulin, in addition to acting as a direct antioxidant and free radical scavenger [66]. Primary receptors for melatonin include the G-coupled transmembrane receptors MT1 and MT2 which are widely expressed throughout the human body as described in Table 1 [67–76].

**Table 1:** Melatonin Binding Sites MT1 and MT2 and known locations for protein exprssion

Binding site	Other Names	Gene location	Central locations for protein expression	Peripheral locations for protein expression
MT1	Mel1a or ML1A	Chromosome 4 (4q35.1)	SCN, amygdala, hippocampus, hypothalamus, nucleus accumbens, substantia nigra, and the cerebellum.	The cardiovascular system and immune system as well as the testes, ovaries, skin, liver, gallbladder, kidney, adrenal cortex, placenta, breast, pancreas, and spleen.
MT2	Mel1b or ML1B	Chromosome 11 (11q21-q22)	Retina, SCN, hippocampus, and cerebral cortex.	Blood vessels, testes, kidneys, gastrointestinal tract, mammary glands, adipose tissue, and the skin.

A third binding site with lower affinity to melatonin compared to MT1 and MT2 was described in 1988 and dubbed MT3 (ML-2), however, it was later discovered that MT3 was in fact the previously characterized enzyme, quinone reductase 2 (QR2), a protein with detoxifying properties [77–79]. It is of note that melatonin binding sites tend to be expressed in low densities throughout various tissues which may be due to the high affinity of melatonin for its receptors [72].

Melatonin may also activate certain nuclear receptors in the retinoid orphan receptor (ROR), or retinoid Z receptor (RZR), family, however, whether melatonin interacts with these receptors directly remains uncertain [80, 81].

In order for melatonin to perform intracellular actions, it must be available intracellularly. A study by Hevia et al. found that members of the SLC2/GLUT family glucose transporters contribute to melatonin uptake. This has potential consequences including that high blood glucose concentrations may impact a cell’s ability to take up melatonin by acting as a competitive ligand of GLUT1. On the other hand, melatonin may reduce the uptake of glucose to cells including tumor cells, as suggested by a study performed in prostate cancer cells [82].

### Physiological effects

Melatonin is a chronobiotic molecule which synchronizes a vast variety of central and peripheral biological functions as an adaptation to the 24-h day-night cycle by binding to the aforementioned receptors. In the absence of light, signals from the SCN are relayed to the pineal gland and stimulate the secretion of melatonin. Melatonin then travels to the SCN and binds to MT1 and MT2 receptors where MT1 activation leads to an inhibition of neuronal activity which normally promotes wakefulness and MT2 activation causes a phase shift in circadian firing rhythms [83, 84]. A recent study conducted in mice demonstrated that the activation of MT1 receptors primarily regulate rapid eye movement (REM) sleep while the activation of MT2 receptors selectively increases non-rapid eye movement (NREM) sleep [85]. Whilst melatonin is most well-known for its regulation of the sleep-wake cycle, it also plays an important role in neural development, blood glucose regulation, immune system function, sexual maturation and the cardiovascular system [86–96].

#### Neurodevelopment

Melatonin plays a significant role in human development in utero where the hormone exhibits effects on placenta function and early nervous system development. Niles et al. demonstrated in vitro that melatonin activates glial cell-line derived neurotrophic factor (GDNF) and that neural stem and progenitor cells express MT1 receptors [86]. These results suggest a potential neuroprotective role of melatonin and may explain why sleep deprivation has been found to suppress neurogenesis in the mouse hippocampus while melatonin supplementation rescues these deficits [97].

#### Reproduction and sexual maturation

The process of sexual maturation may also be impacted by melatonin [87]. Existing evidence indicates that the decrease in nocturnal melatonin levels during adolescence correlates to sexual maturation. This is further supported by the inverse relationship between levels of luteinizing hormone and melatonin at various stages of puberty and the decline in salivary melatonin levels with advancing tanner stages in both boys and girls [56, 88]. There has been some concern that long-term exposure to exogenous melatonin may alter the timing of puberty onset in adolescents as seen in some animal studies, however more research is needed before drawing similar conclusions in humans [98, 99].

#### Cardiovascular system

Beneficial effects of melatonin on various cardiovascular risk factors such as hyperlipidemia, hypertension, and obesity have been demonstrated in numerous studies [89]. Celinski et al. demonstrated that melatonin supplementation at 5 mg twice a day for 14 months significantly lowered LDL cholesterol and triglyceride levels in patients with nonalcoholic fatty liver disease [90]. Supporting this, Scheer et al. demonstrated that melatonin supplementation may lower blood pressure in patients with untreated essential hypertension [91]. In keeping with these findings, a study performed in rats by Simko et al. showed that continuous light exposure can lead to hypertension, left ventricle hypertrophy and fibrosis, as well as increased oxidative stress in the left ventricle and aorta [100].

#### Immune system regulation

It is hypothesized that one of melatonin’s key roles throughout evolution involved protecting organisms from environmental stressors by acting as a potent antioxidant [50]. Melatonin detoxifies numerous reactive oxygen and nitrogen species including hydrogen peroxide, hydroxyl radical, singlet oxygen, peroxyl radicals, and nitric oxide radicals. Several in vivo studies have found melatonin to be a more effective antioxidant than the well-known vitamin C and vitamin E in terms of protection from tissue damage [92, 93]. In addition to acting as an antioxidant itself, melatonin upregulates the expression of anti-oxidative enzymes such as Cu/Zn-superoxide dismutase (CuZn-SOD), Mn-superoxide dismutase (Mn-SOD), catalase, and glutathione peroxidase (GPx) [94]. The hormone also enhances T- and NK- cell responses in addition to stimulating the production of cytokines and interleukins including IL-2, IL-6, and IL-12 [95, 96].

In uveal melanoma, the occurrence of micrometastases is dependent on a delicate balance between supportive and inhibitory interactions between the tumor cells, the surrounding stroma and the immune system [29, 101, 102]. While response rates are low for CTLA4 and PD-L1 immune checkpoint inhibition in metastatic uveal melanoma, other immune checkpoints are at play and increasing the T- and NK- cell response in the metastatic niche may inhibit both survival and growth of uveal melanoma micrometastases [103].

#### Melatonin and mitochondria

Melatonin is an effective antioxidant in mitochondria. Mitochondria synthesize melatonin locally in addition to performing active take-up from cytosolic pools derived from systemic circulation [104–106]. Melatonin counteracts oxidative stress and promotes the activity of enzymes involved in oxidative phosphorylation, increasing ATP production in neuronal and hepatic mitochondria in rats [107]. Yamamoto et al. demonstrated that melatonin reversed the deadly effects of cyanide, a complex IV inhibitor, in mice [108].

#### Blood glucose

Melatonin plays a role in the regulation of blood glucose levels via its association with MT1 and MT2 [109]. When melatonin binds MT1 and MT2 receptors present in the pancreas, insulin secretion is reduced via a receptor mediated inhibition of the cyclic adenosine monophosphate (cAMP) and cyclic guanosine monophosphate (cGMP) pathways [110, 111]. On the other hand, activation of MT1 and MT2 receptors on pancreatic alpha cells has been shown to increase glucagon secretion via Gαq-coupled and PI3K signalling pathways [112]. Further supporting the role of melatonin in blood glucose regulation, studies have found that pinealectomized rats display an altered daily rhythm in glucose levels with increased glucose concentrations at night and mice lacking MT1 receptors entirely have higher overall mean blood glucose levels [113, 114].

### Therapeutic effects

Previous research indicates a therapeutic benefit of melatonin for multiple types of cancer. In patients with advanced and metastatic non-small cell lung cancer were treated with 10 mg of oral melatonin in a cycle consisting of 21 treatment days followed by 7 rest days, as compared to supportive care alone, there was a significant increase in both one-year survival and disease stabilization without drug-related toxicity [115]. In another study, melatonin had a small therapeutic effect on stage 3 and 4 human metastatic malignant melanoma and with few side effects despite a relatively high oral dose of 50 mg 4 times a day for a minimum of 2 months [116]. Lissoni et al. demonstrated that in patients with metastatic solid tumors, including non-small cell lung cancer, breast cancer, gastro-intestinal tract tumors as well as head and neck cancers, there was a higher survival rate and a longer mean time to progression for patients treated with 20 mg oral melatonin 7 days prior to chemotherapy compared to those treated with chemotherapy alone. This is potentially indicative of the antioxidative effects of melatonin which may help mediate chemotherapy toxicity and even enhance of the efficacy of chemotherapies on cancer cells [117]. Oral melatonin at 40 mg daily amplifies the anti-tumor activity of IL-2 treatments in patients with solid neoplasms when given 7 days before IL-2 is administered [118]. In glioblastoma patients treated with radiotherapy in addition to 20 mg oral melatonin daily until disease progression, there was an increase in one-year survival rate as well as lower therapy-related toxicity compared to radiotherapy alone [119]. When adding 20 mg of melatonin daily to 14 women with metastatic breast cancer who did not respond to tamoxifen therapy alone or progressed after an initially stable disease status, a partial response was achieved in four participants (29%). Mean serum levels of the breast cancer growth factor IGF-1 and patient anxiety levels were significantly decreased [120].

An in vitro study on endometrial adenocarcinomas demonstrated that a physiological concentration of melatonin at 10^− 9^ M for 4 h and 96 h incubation showed no inhibition on SNG-II estrogen receptor-negative, endometrial cancer cell growth. However, melatonin at the same concentration had a significant inhibition effect on Ishikawa estrogen receptor-positive endometrial cancer cell growth [121]. In ovarian adenocarcinoma BG-1, treatment with melatonin at a concentration of 10^− 9^ – 10^− 7^ M for 48 h resulted in a 20–25% reduction in the number of cancer cells in vitro [122].

Several studies have shown that melatonin is beneficial when used in conjunction with other anti-cancer drugs and radiation treatments by reducing their toxicity on healthy human cells while enhancing the drugs’ effect on cancer cells [123–128]. This may be due to the antioxidative properties of Melatonin which neutralizes toxic hydroxyl radicals responsible for cell damage at the nucleic acid level, protecting from ionized radiation as well as from single- and double- stranded breaks in DNA, offering resilience to DNA modifications in healthy cells [129]. At the same time melatonin’s enhancement of the efficacy of chemotherapy drugs may be related to its pro-apoptotic features [130]. A review by Wang et al. found that Melatonin decreased radiochemotherapy-related side effects such as thrombocytopenia (Relative risk, RR = 0.13), neurotoxicity (RR = 0.19), and fatigue (RR = 0.37) in patients with solid tumors including [125]. Another study found that melatonin treatment protects bone marrow when used prior to whole body radiation or when combined with proteasome-inhibitor drugs such as Bortezomib, melatonin reduces the drug’s toxic effects [131]. Similarly, when combining cisplatin with melatonin in the treatment of ovarian cancer melatonin protected normal ovarian cells from cisplatin’s toxicity [132, 133]. Furthermore, melatonin supports the apoptotic effects of Doxorubicin when both are applied to human breast cancer cells [132].

Several in vitro studies suggest that the concentration of melatonin used may impact its effects on malignancies. For example, a study conducted using rodent melanoma cells by Slominski et al. demonstrated that low concentrations of melatonin (0.1–10 nM) inhibited cell proliferation without impacting melanogenesis while high concentrations (> 0.10 μM) inhibited melanogenesis while having no effect on cell proliferation and that particularly high concentrations (100 μM) were found to stimulate cell proliferation [134]. Similarly, an in vitro study utilizing two cell sublines of mouse melanoma found that a melatonin concentration of 1 nM significantly inhibited cell proliferation while sub-physiologic concentrations (0.1 pM) and supraphysiologic concentrations (100 nM and 10 μM) had no impact [135]. Fisher et al. demonstrated that melatonin suppressed cell proliferation at all tested concentrations (1 mM – 100 nM) in four different human melanoma cell lines [136]. These studies suggest that further research is required to assess the dose dependent actions of melatonin in inhibiting cancer cell proliferation.

### Oncostatic effects

**Fig. 4:**
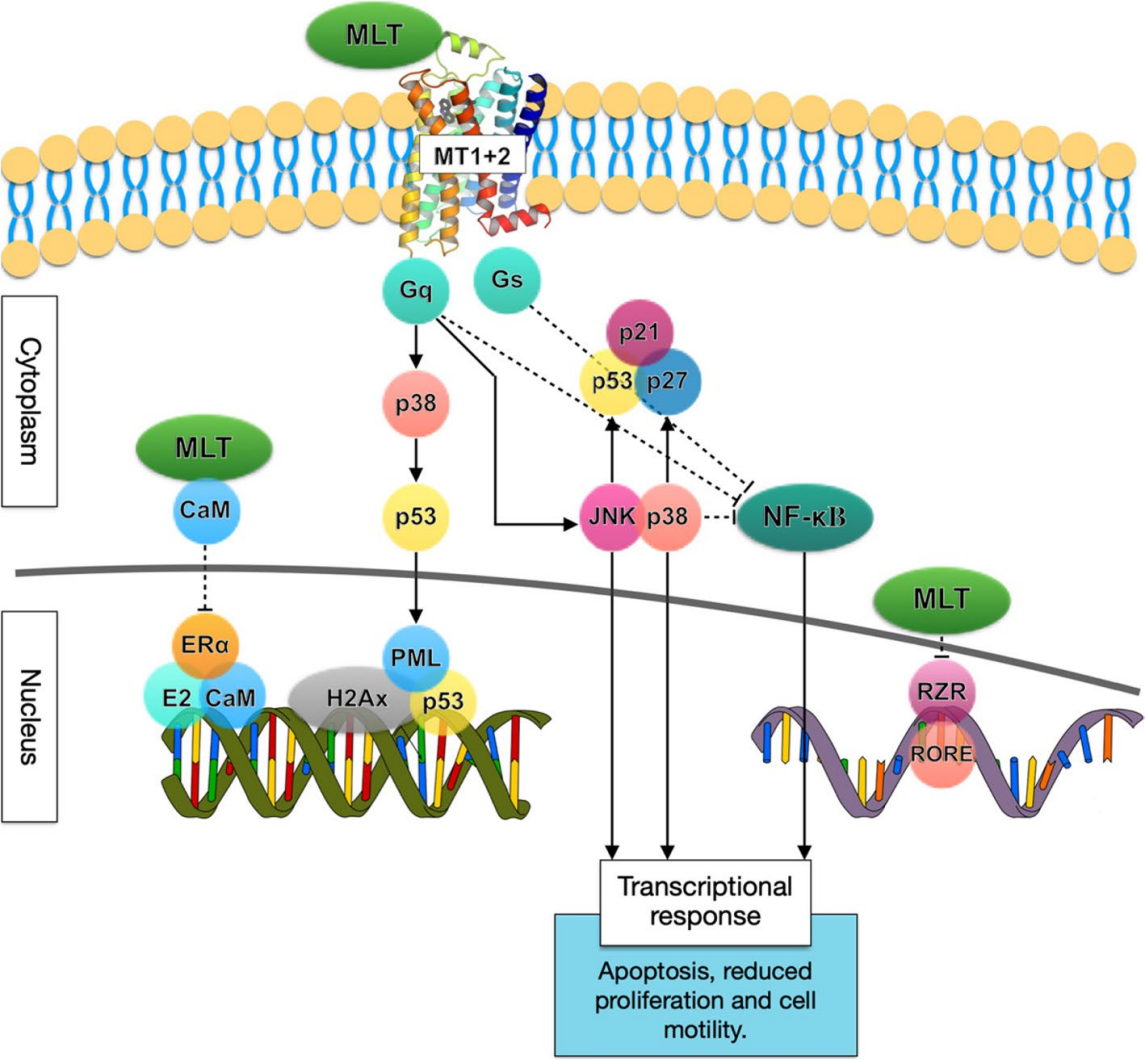
Oncosuppressive mechanisms mediated by melatonin. Melatonin (MLT) activates the high-affinity G protein-coupled receptors MT1 and MT2 which reduce the transcriptional activity of NF-κB and activates phosphorylation cascades mediated by mitogen-activated protein kinases (MAPKs) including MEK1/2, ERK1/2, JNK, and p38. In turn, NF-κB inhibition and MAPKs activation inhibit cell growth and motility and promote apoptosis and DNA damage repair through accumulation of oncosuppressors such as p53, p27kip1, and p21. NF-κB inhibition and MAPKs activation also activates DNA repair complexes such as P53/PML/H2AX on DNA damage sites, and transcriptional control of genes involved in the cell cycle, apoptosis, and invasiveness. Further, melatonin can bind to the intracellular protein calmodulin (CaM) and reduce the Estrogen Receptor α (Erα) response in ER positive cells by impairing the formation of a proper E2–Erα–CaM complex on target genes. Melatonin downregulates the nuclear RZR receptors (RZR alpha, RZR beta, ROR alpha 1, RZR alpha 2, ROR alpha 3 and ROR gamma), inhibiting growth, angiogenesis and HIF-1α activity. Arrows indicate activation, while dashed blunt lines indicate inhibition. Activation indicates an increase in protein or activity levels, while inhibition indicates a decrease in protein or activity levels

In addition to melatonin’s immunoenhancing features and its synergistic effects when used in conjunction with anti-cancer drugs, it possesses several oncostatic properties. A meta-analysis published in 2005 evaluated the effects of melatonin in cancer treatment where melatonin was either used as the sole treatment or as an adjuvant therapy for patients with solid tumors. Ten randomly controlled trials were reviewed, and results showed an overall 44% reduction in the risk of death at 1 year [63]. Oncosuppressive mechanisms mediated by melatonin are summarized in Fig. 4.

#### Apoptosis and tumor invasion

Melatonin is involved in cell cycle regulation, apoptosis induction, and metastasis inhibition. A proposed mechanism for these processes involves melatonin’s inhibition of NF-kB and activation of MAPKs which suppresses cell growth and motility in addition to promoting apoptosis through an accumulation of oncosuppressors such as p53, p27kip1, and p21 [137]. NF-κB inhibition and MAPKs activation also activates DNA repair complexes which help prevent the accumulation of DNA mutations caused by chemotherapeutic agents or ionizing radiations [138]. Melatonin has also been shown to inhibit cancer cell migration by reducing the expression of proteins involved in tumor cell progression and metastasization [137, 139–141]. An in vitro and in vivo study by Borin et al. found that melatonin decreased the expression of the Rho-associated kinase protein (ROCK-1), a protein associated with tumor growth and metastasis [141, 142]. Results from an in vitro study by Zhou et al. suggest that melatonin increases the expression of occludin, a regulatory protein in tight junctions where decreased expression has been associated with cancer and increased tumor invasion [139, 143].

#### Angiogenesis

Tumors with high levels of vascular endothelial growth factor (VEGF), an endogenous pro-angiogenic protein, often grow faster and metastasize earlier resulting in poor prognosis [144, 145]. Melatonin has been found to have direct effects on angiogenesis, through inhibitory actions on VEGF as well as indirect anti-angiogenic effects through the suppression of tumor growth factors and the neutralization of ROS which normally stimulate angiogenesis in tumors [146–148]. Further, via downregulation of the nuclear RZR receptors (RZR alpha, RZR beta, ROR alpha 1, RZR alpha 2, ROR alpha 3 and ROR gamma), melatonin has been shown to inhibit tumor cell HIF-1α activity and angiogenesis both in vitro and in vivo [149, 150]. Inhibition of HIF-1α has previously been shown to reduce both primary tumor growth and development of macrometastases of uveal melanoma [151].

#### Anti-estrogenic

Other oncostatic effects of melatonin include its anti-estrogenic properties which may contribute to the positive results of melatonin supplementation on patients with hormone dependent tumors as seen in certain breast, ovarian, and prostate cancers [105]. Recent research indicates that there is also increased estrogen receptor expression in uveal melanoma with poor prognostic features [152]. Melatonin regulates estrogen receptor expression by inhibiting the binding of the E2-ER complex to the estrogen response element (ERE) at the DNA level [153–155]. Another potential mechanism involves inactivation of calmodulin, a modulator of Erα transcription [156–158].

### Cell lines

A study published in 1997 investigating the effects of melatonin on human uveal melanoma cells in vitro demonstrated that melatonin can suppress the growth of the cancerous cells with a mean inhibition rate of 50% for concentrations of 0.1–10 nM [159]. A year later, Hu et al. tested the effects of various derivatives and precursors of melatonin on the growth of cultured human uveal melanoma cells. These results demonstrated that melatonin can inhibit the growth of the cells relative to the given dose with concentrations ranging from 0.1 to 10 nM with 41% inhibition compared to untreated samples [160]. Note that the concentration of melatonin found in human aqueous humor is ~ 2 nM. In addition, they found that a derivative of melatonin, 6-chloromelatonin, was an even more potent growth inhibitor (47% inhibition compared to untreated samples) whilst melatonin’s precursors tryptophan and serotonin failed to inhibit cell growth over the same concentration ranges [160]. Furthermore, uveal melanoma cells have been found to express transmembrane receptors for melatonin, and a study by Roberts et al. found that melatonin and its membrane receptor agonists (MT1 and MT2) inhibited growth of uveal melanoma cells at low concentrations while having no impact on the growth of normal, non-cancerous, melanocytes [161].

### Animal studies

Animal studies investigating the effects of melatonin in cutaneous melanoma have found mixed results. In one mouse study, murine melanoma model B16-F10 cell-line were injected in the tail vein of melatonin knockout C57BL/6 J mice which were then treated with melatonin 20 mg/kg as an intraperitoneal injection or in drinking water. After 15 days, there were no significant differences compared to controls (not receiving melatonin) in the number and size of lung metastases at the micro- or macroscopic level [162]. Another study investigated the effect of melatonin analogues on a human melanoma xenograft mouse model where eight-week old mice were injected with DX3 melanoma cells that were allowed to grow for 1 week in vivo. The study showed that subcutaneous injections of UCM 1033 and UCM 1037 melatonin analogues inhibited tumor growth by 40 and 90% respectively, while melatonin provided no significant tumor growth suppression when measured both 4 weeks after treatment and after euthanization [163]. Melatonin treatment assessed in athymic male and female mice which received 5 μg/g melatonin in drinking water daily at the time of B16 melanoma inoculation, showed reduced melanoma growth size and weight compared to control groups 40 days after administration [164]. An additional study examined male and female athymic mice that were injected with 7 × 10^4^ melanoma cells and received either 5 μg/g body weight of melatonin in drinking water daily with 0.5% ethanol or only 0.5% ethanol or none of the above. Compared to the control group, the growth of B16 melanoma was less in melatonin-treated mice. At day 40, melatonin-treated mice had smaller tumors in both size and weight [164].

### Human studies

In the previously mentioned meta-analysis by Mills et al., treatment with melatonin was associated with a 44% reduction in the risk of death at 1 year in human patients with solid tumors, suggesting that the therapeutic effects of melatonin seen in cell lines and animals likely extend to humans as well [63]. A specific clinical trial examined the effects of melatonin in 40 patients with advanced malignant melanoma including 10 patients with ocular melanoma [165]. The participants were given oral melatonin with doses ranging from 5 to 700 mg/m^2^ four times a day with a median follow-up time of 5 weeks. Twelve patients showed a response or stable progression of disease depending on the dose, type of melanoma and metastasis location, including the CNS, lymph nodes, liver and lungs. Doses at 75 mg/m^2^ showed a more extensive response with minimal side effects, which were manifested primarily as fatigue. An interesting finding in this study is the response observed in the brain as two patients with brain metastases had a response to melatonin, which can cross the blood brain barrier. This is noteworthy as other current standard treatments are ineffective in this region [165]. Another clinical trial studied the effect of adjuvant 50 mg melatonin administered orally in five patients with metastatic melanoma. One patient remained on therapy for > 8 months and had almost total regression, whilst the other four had no objective response to melatonin [166].

Recently, a large, randomized double-blind placebo-controlled clinical trial for patients with non-small cell lung cancer (NSCLC) reported noteworthy results [167, 168]. In an adjuvant setting, 709 participants were randomized to 20 mg melatonin or placebo at night for 1 year following surgical resection of primary NSCLC. The primary endpoint of two-year disease-free survival (DFS) was not met (RR 1.01, 95% confidence interval (CI) 0.83 to 1.22). In a subgroup analysis however, a hazard reduction of 25% (hazard ratio 0.75, 95% CI 0.61 to 0.92) in 5-year DFS was found for patients with more advanced stage (III/IV) at surgical resection [167]. Similarly, patients with stage IIIb or IV NSCLC receiving 20 mg melatonin at night in another randomized double-blind placebo-controlled trial had an all-cause mortality hazard reduction of 39% (HR 0.61, 95% CI 0.39 to 0.96) [167].

### The potential for melatonin as an adjuvant treatment in uveal melanoma

Melatonin possesses oncostatic properties and has shown promising results as an anti-cancer therapy in various types of cancers [30–33]. As suggested by studies of uveal melanoma- and normal uveal melanocyte cell lines, melatonin may selectively inhibit the growth of the former by activation of the melatonin Mel1b membrane receptor [161]. Other observations of cell lines as well as animal studies suggest that both melatonin as well as its derivatives and analogues inhibit the growth of both uveal and cutaneous melanoma cells [160, 163, 164]. An additional benefit of melatonin specific to uveal melanoma patients could be related to its antioxidative features which may help counteract the negative ocular side effects of radiation in patients treated with plaque brachytherapy [124]. Of interest is that some studies have found that the hormone’s derivatives and analogues are more potent in their anti-cancer effects than melatonin itself which should be considered in future clinical trials [163]. Other studies found no significant impacts of melatonin on cancer cell growth [162]. Factors which may influence these results include the dose or concentration of melatonin used, the study duration, the population size as well as the stage of disease [162].

As seen in randomized clinical trials including patients with NSCLC, melatonin may have a greater effect on more advanced stages of cancer [167, 168]. This is of special relevance to uveal melanoma, considering the tendency for early seeding of micrometastases [169]. Previous studies demonstrate that the seeding of these metastasis to other organs, primarily the liver, often occur before the detection of the primary tumor [27, 28]. The micrometastases can then remain dormant for several years before proliferating and developing into macrometastases [29]. When primary uveal melanoma is diagnosed in patients, the cancer could be considered to already be in an advanced stage. Melatonin pocesses a broad spectrum of qualities desired in an adjuvant treatment such as general oncostatic, immunoenhancing, anti-oxidative, and anti-angiogenic properties while also possessing anti-metastatic properties relevant to more advanced cancer stages. Furthermore, the incidence of uveal melanoma peaks in patients over 60 years, which coincides with an age-related reduction in endogenous melatonin production [56, 59]. Thus, the herein reviewed anti-cancer effects of melatonin supplementation could potientially be greater in this age group than in younger populations.

Most human studies have provided participants with doses ranging from 10 to 40 mg, including the studies analysed in the systematic review by Mills et al [63]. Twenty milligram at night has been selected for no less than 13 randomized cancer trials [119, 120, 167, 168, 170–178]. This dose range may therefore be a good starting point for a randomized trial with adjuvant melatonin for patients with uveal melanoma.

## Discussion

In this review, we have explored the rationale for using melatonin as an adjuvant treatment for non-metastatic uveal melanoma. This cancer is characterized by a relatively high proportion of patients developing metastatic disease even when the eye containing the primary tumor is immediately enucleated, with up to 50% of patients developing metastases within 25 years [22, 179]. This trend has been attributed to the spreading of clusters of dormant tumor cells called micrometastases to distant organs, notably the liver. By the time these micrometastases grow large enough to be detected, prognosis is fatal with a median patient survival time of less than 1 year [180]. Consequently, micrometastases and dormancy are central to the progression of uveal melanoma. The concept of dormancy here can be described as a phenomenon where malignant cells enter the G0 phase of the cell cycle and experience a state of temporary mitotic arrest. These so called micrometastases, however, maintain the potential to reactivate after several years or even decades and begin to grow, as seen in uveal melanoma [181]. It is therefore vital in the treatment of uveal melanoma to inhibit the development and migration of these micrometastases as soon as possible upon detection of the primary tumor.

Melatonin has been studied for several decades and has been shown to suppress the growth of cancer cells by up to 50% in vitro [159] and melatonin analogues have been found to suppress the growth of cancer cells by up to 90% in vivo [163]. Despite the limited number of studies investigating uveal melanoma in humans, a positive response to treatment with melatonin on cancer progression and disease stability has been observed in other types of cancer at various doses [165]. Melatonin has also been found to increase the efficiency of other standard cancer treatments such as chemotherapy and radiation in addition to possessing oncostatic properties itself [123–128]. As discussed, melatonin enhances our immune system, stimulates apoptosis in cancerous cells, inhibits angiogenesis, and regulates estrogen receptor expression. Therefore, it may be regarded as a suitable candidate for patients with uveal melanoma, especially in cohorts with non-metastatic uveal melanoma but high risk of later metastatic progression [179].

In previous studies, treatment with melatonin has been associated with a higher survival rate, longer mean time to progression, and a better response cytotoxic chemotherapy with less drug-related toxicity [117–120]. In uveal melanoma, early micrometastasis migrate via the blood stream to other organs and are usually detected long time after the primary tumour diagnosis [23, 25, 27, 28]. In this sense, uveal melanoma might therefore be regarded as an advanced systematic disease at onset, for which adjuvant treatment regimes are undoubtedly needed. Approximately 50% of patients with uveal melanoma who develop metastases, do so within 5 years from diagnosis, therefore a clinical trial investigating the impact of melatonin as an adjuvant treatment would require continuous supplementation over an extended period of time [179]. Such a project is motivated given the few reported side effects of melatonin, which include mild symptoms such as light diarrhea, drowsiness, fatigue, and itching without any significant effects on sexual health, sleep or mental status [116]. In summary, the characteristics of uveal melanoma taken together with the oncostatic properties of melatonin, support the importance of conducting a clinical trial with melatonin as an adjuvant treatment in newly diagnosed patients.

### Limitations

There are limitations to this review which should be taken into consideration. First, no studies were found which evaluated the effects of melatonin at high doses for a long period of time in humans. Second, while studies were found which observed anti-metastatic properties of melatonin in vitro and in animal subjects, no studies were found which specifically investigated metastasis prevention in human subjects. Third, this systematic review relies on a limited number of databases where few studies focusing specifically on uveal melanoma were found.

With regards to potential limitations in the use of melatonin as an adjuvant treatment, one might argue that there isn’t enough regulation at present as melatonin is widely accessible and in some countries such as the United States can be bought at grocery stores without a prescription. This may pose challenges for controlling the use of the molecule in patients. While this issue may have to be addressed in the future, melatonin has been found to be a particularly safe supplement with few side effects which are relatively mild.

## Conclusions

Uveal melanoma is a rare but serious form of cancer with a high rate of metastasis which can appear years to decades after the primary tumor is identified and removed. Results from both animal and human studies suggest that melatonin has the potential to suppress the growth of malignant cells and inhibit the development of metastasis in numerous types of cancers. This may be particularly beneficial in uveal melanoma where protection from the development of micrometastasis is vital immediately upon diagnosis as the prognosis diminishes significantly with the detection of metastases. Animal and cell line studies suggest that melatonin may have these oncostatic effects on uveal melanoma, however, no clinical trial specific to uveal melanoma has been performed on human patients. Future studies investigating the impact of long-term melatonin supplementation in patients diagnosed with uveal melanoma is the next step in this investigation.

## Data Availability

Data sharing is not applicable to this article as no datasets were generated or analyzed during the current study.
